# In search of a happy medium for preterm infant respiratory support at birth:

**DOI:** 10.1038/s41390-025-04513-z

**Published:** 2025-10-22

**Authors:** Kurlen Payton, Manoj Biniwale, Rangasamy Ramanathan

**Affiliations:** Cedars Sinai Guerin Children’s, Cedars Sinai Medical Center, Los Angeles, CA USA

## Abstract

**Key Messages:**

- Optimal CPAP during transition is not well established
- Higher CPAP and supplemental Oxygen improves transition in a preterm animal model

**Impact:**

- Clinicians will likely consider using higher CPAP during respiratory transition in the delivery room
- Use of a higher Oxygen than currently recommended and titration of CPAP and Oxygen based on the response may improve the transitional issues

The story of the emperor and the moth is an allegory that may be relevant to delivery room (DR) management in preterm infants with respiratory distress. In the story, an emperor watches a moth struggle to escape from a cocoon. The emperor patiently waits for the moth to emerge. Ultimately, he becomes impatient. He cuts the cocoon open to free the struggling moth. This seems ironically similar to an elective caesarean section. Unfortunately, his well-intended support had unintended consequences. The moth struggled after exiting the cocoon and was not strong enough to fly. The moth dies because the natural process of the struggle to exit the cocoon did not occur. The process, surprisingly similar to transitional respiratory physiology, involves fluid moving from the body into the moth’s wings. This is a beneficial developmental process that prepares the moth for life outside the cocoon. The neonatal community is faced with a similar dilemma in finding the optimal amount of support in the delivery room for preterm infants. We want to provide the appropriate amount of support while avoiding unintended consequences of too much support. Ironically, as with the moth, fluid shifts play a leading role in this DR drama.

The full-term infant usually establishes functional residual capacity (FRC) as alveolar fluid moves through the interstitium into lymphatics and pulmonary circulation soon after delivery, as a first example of the newborn’s autonomy outside the womb. However, preterm infants are unable to conquer this transition from a fluid-filled lung to an air-filled lung without additional support.^1,2^ Varying degrees of surfactant deficiency, weak intercostal muscles, inadequate diaphragm strength, reduced respiratory drive, intermittent glottic closure, and airway collapse prevent the desired clearance of alveolar fluid into the interstitial compartment. This ultimately leads to poor oxygenation and ventilation. Historically, the field of neonatology has erred on the side of aggressive invasive respiratory support. Seminal studies have repeatedly helped the field to consider a “less is more” approach to respiratory management. In the 1970’s, non-invasive strategies aimed at treating respiratory distress syndrome (RDS) emerged.^3^ The COIN trial further helped us find balance showing that a trial of continuous positive airway pressure (CPAP) of 8 cmH_2_O was non-inferior to intubation and surfactant immediately after birth; however, use of high CPAP pressure resulted in a higher incidence of pneumothorax (9% vs 3%).^4^

The current evidence-based standard is to avoid tracheal intubation in the DR at birth by using non-invasive ventilation. There may be more nuanced approaches to improve care with non-invasive support. This concept of determining “how much to help” is the primary dilemma in the management in preterm infants with respiratory distress. Providing right amounts of pressure in a timely fashion may improve outcomes of preterm infants. Inadequate pressure delivery in the DR can lead to delay establishing FRC leading to respiratory failure. Higher pressures provided through CPAP can be detrimental to the lungs causing barotrauma, volutrauma, and abdominal distention preventing lung expansion. Historically, the lessons of the emperor and the moth story play out in the NICU. More recent evidence shows that preterm infants administered CPAP while spontaneous breathing compared to positive pressure ventilation were shown to have better respiratory function and plethysmography amplitude gain. This example highlights the delicate balance of finding the happy medium for providing optimal respiratory support.

Study by Cannata et al. helps us to consider how clinical practice may evolve in providing CPAP and oxygen in the DR.^5^ They report the effects of two CPAP levels and two amounts of oxygen delivery on spontaneous breathing and lung inflation in a preterm rabbit model. In this study, prematurely delivered rabbits were administered 4 different combinations of levels of CPAP and FiO_2_. Breathing rates, amount of lung aeration, lung bulging, and air accumulation in the stomach were measured to determine the impact of different CPAP and FiO_2_ combinations. CPAP was delivered at pressures of either 5 cmH_2_0 or 15 cmH_2_0. The FiO_2_ was kept at either 0.30 or 0.60. The model of the study addresses everyday dilemma clinicians face in the DR while providing respiratory support to preterm infants. The lower CPAP (5 cmH_2_0) and lower FiO_2_ of 0.30 studied is similar to current neonatal resuscitation program recommendations for preterm infants.

The authors used custom-made ventilators with CPAP mask to provide accurate support with CPAP. A vibrotactile stimulation device was used to prevent disrupting imaging. Phase contrast x-rays used in this study provide unique advantage by enhancing refractive index between air and tissue helping to calculate lung volumes. This assessment provides real-time objective assessment of the impact of various ranges CPAP and FiO_2_. The authors found association of higher CPAP with better lung aeration whereas rabbit kittens receiving higher FiO_2_ had higher spontaneous breathing rates. The preterm rabbits receiving both the higher CPAP and higher FiO_2_ benefitted the most. The authors note that CPAP and FiO_2_ appeared to provide support for FRC and breathing rates independent of each other. It is interesting to note that higher FiO_2_ had better impact on breathing rate than level of CPAP which authors attribute to hypoxia being a potent inhibitor of spontaneous breathing in newborns. Higher FRC was directly related to CPAP level which is plausible for opening of stiff alveoli and moving alveolar fluid into the interstitial space. CPAP belly and lung bulging are of concern when high CPAP is provided. It is reassuring to see CPAP of 15cmH_2_O did not lead to any of these adverse consequences. The results are also notable in that the standard respiratory support currently used in preterm infants led to worse outcomes.

Results from this study as well as evidence from other pre-clinical and clinical studies suggest opportunities for more nuanced DR support.^6^ The study raises two important concepts that may be vital for evidence-based progress in optimal DR respiratory management. First, real-time individualized and “responsive” CPAP may be optimal as opposed to a single pressure target over time. Secondly, oxygen delivery may be better used to target real-time physiological changes as opposed to a predefined oxygen saturation range. The study highlights that CPAP and oxygen delivery may target separate pathophysiologic mechanisms that fluctuate in degree of severity during the transitional period. This complex and changing physiology calls for more complex and variable management strategies rather than having rigid guidelines for all preterm infants.

The respiratory transition occurs in three distinct phases (Fig. 1).^7^ First, fetal lung fluid must be removed from alveoli. Secondly, there needs to be enough distending pressure to push against the hydrostatic pressure of the fluid that moved into the tissue surrounding the alveolus. This pressure may also help prevent reentry of the fluid. Lastly, the alveoli are ready for optimal oxygenation and ventilation, presumably with less of a fluid burden. Each one of these phases may require different pressures for keeping alveoli open leading to effective gas exchange. This is complicated by the fact that every baby’s need may be changing over the first few minutes of life. Higher CPAP earlier in the transition makes sense acknowledging the three phases of progression from fluid filled alveoli to alveoli ready for gas exchange. Presumably the highest CPAP would be needed soon after birth and could be titrated down to meet the needs along a continuum. In contrast, there are studies suggesting that CPAP pressures above the current standard may not provide additional benefit. Studies suggest that CPAP > 8-9 cmH_2_O may have reduced benefit and may cause harm.^4,8^ The varying results in these studies may be due to an inability to tease out varying physiologic changes that occur in the minutes and hours after delivery.

**Fig. 1:**
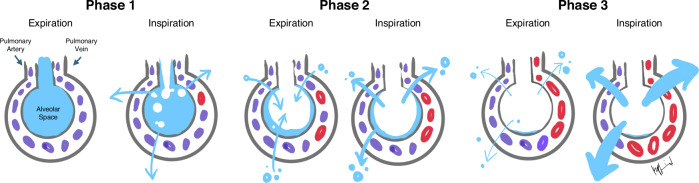
Optimal CPAP level and FiO_2_ may vary across the three distinct phases of newborn lung fluid shifts with changes during inspiration and expiration during respiratory transition after birth.

The study suggests that oxygen could be viewed more as a respiratory stimulant and administered accordingly. Target oxygen saturation is typically determined using the NRP algorithm and oxygen delivery is titrated accordingly. It’s not clear of the saturation targets that are optimal during each of the transitional phases. Preterm infants tend to hang on to their fetal physiology with intermittent closure of the larynx that was previously serving the role as the door preventing fluid from exiting the lungs. This leads to apnea which compromises the transition and prevents progression through the three phases. Tactile stimulation is used to address this in the DR. Perhaps a more proactive oxygenation strategy aimed at improving spontaneous breathing efforts could help support the preterm infants’ transition more effectively.^8,9^

The approach of receiving high CPAP and oxygen delivery though promising must be approached with caution especially in extremely preterm infants. Current NRP recommendations are not just meant for improving breathing and lung aeration in the DR. They also attempt to address both short- and long-term consequences in these vulnerable infants. Previous studies with aggressive use of oxygen and ventilation in the DR have shown increased risk for BPD and mortality. Studies involving sustained inflation, providing high CPAP levels for a short amount of time in the DR, did not show significant benefits.^10^ Recent meta-analysis of studies involving high FiO_2_ for neonatal resuscitation in preterm infants did reveal decrease in mortality though quality of evidence remains low.^11^

To translate findings of the present study in the real world, real-time monitoring is needed. Lung physiology can be imaged and measured.^12,13^ Non-invasive ventilation could be supplemented with NAVA, electrical impedance tomography, point of care ventilation to perfusion assessment, lung ultrasound, lung near infrared spectroscopy or other real-time assessment tools.^14–17^ Titrating FiO_2_ and CPAP in real time based on these assessments may be feasible. However, it is currently not well studied but could be achieved in the near future using artificial intelligence. Studies exploring responsive CPAP approaches are ongoing and the challenges of this approach are being conducted.^6,18^

In summary, Canatta et al. have shown in this animal model that use of higher CPAP and higher FiO_2_ with titration of both helps to improve respiratory transition.^5^ A collaborative approach with the preterm infant being empowered to help themselves where they can, while being given the right pressure and the right time may be the ideal path to further improvements in outcomes. The neonatal community of scientists and clinicians are under pressure to reframe how we approach DR respiratory management in preterm infants.
